# ^18^F-Fluorodeoxyglucose Positron Emission Tomography Combined With CT in Unresolved ICU Infections: Diagnostic Yield and Clinical Consequences in a Retrospective Cohort

**DOI:** 10.1097/CCE.0000000000001467

**Published:** 2026-08-25

**Authors:** Knut Kampe, Theresa Lohse, Susanne Klutmann, Ivayla Apostolova, Gerold Soeffker, Lina Ko, Axel Nierhaus, Stefan Kluge

**Affiliations:** 1 Department of Intensive Care Medicine, University Medical Center Hamburg-Eppendorf, Hamburg, Germany.; 2 Department of Diagnostic and Interventional Radiology and Nuclear Medicine, University Medical Center Hamburg-Eppendorf, Hamburg, Germany.

**Keywords:** critical illness, 18F-fluorodeoxyglucose positron emission tomography combined with CT, infection source identification, intensive care unit, sepsis

## Abstract

**IMPORTANCE:**

In critically ill patients with sepsis, the infectious focus may remain unclear despite microbiology, ultrasound, and conventional cross-sectional imaging. In this setting, ^18^F-Fluorodeoxyglucose Positron Emission Tomography Combined With CT (FDG PET/CT) is used selectively, but its diagnostic value and downstream clinical consequences are not well defined.

**OBJECTIVES:**

To assess the diagnostic yield of FDG PET/CT in selected ICU patients with unresolved infection, describe the infectious foci detected, and document subsequent diagnostic and therapeutic actions.

**DESIGN, SETTING, AND PARTICIPANTS:**

Retrospective single-center cohort study of 108 FDG PET/CT scans performed in 103 adult patients with sepsis treated in the ICUs of a tertiary university hospital. PET/CT was requested either to search for an unknown infectious focus or to confirm or exclude a suspected infectious focus.

**MAIN OUTCOMES AND MEASURES:**

PET/CT findings suspicious for infection, confirmation according to a composite clinical reference standard, classification as clinically informative, diagnostic performance, and diagnostic or therapeutic actions after imaging.

**RESULTS:**

FDG PET/CT detected a subsequently confirmed infectious focus in 72 of 108 scans (66.7%). Eight additional findings suspicious for infection were not confirmed, and one scan was classified as false negative. Apparent sensitivity was 98.6% (95% CI, 96.0–100.0%), specificity 77.1% (95% CI, 63.2–91.1%), positive predictive value 90.0% (95% CI, 83.4–96.6%), and negative predictive value 96.4% (95% CI, 89.6–100.0%). When PET/CT was used to search for an unknown focus, at least one infectious site was subsequently confirmed in 53 of 81 scans (65.4%). When it was used to confirm or exclude a suspected focus, results were diagnostically conclusive in 24 of 27 scans (88.9%). Overall, 77 scans (71.3%) were considered clinically informative. Imaging findings were followed by additional noninvasive diagnostics in 50 scans, invasive diagnostic or therapeutic procedures in 52 scans, and antimicrobial modification in 44 scans.

**CONCLUSIONS AND RELEVANCE:**

In selected critically ill patients with sepsis and unresolved infection, FDG PET/CT often identified a confirmed infectious focus or helped reduce diagnostic uncertainty. These findings support its use as a complementary, problem-oriented diagnostic tool rather than as routine imaging in ICU sepsis. Because referral was selective, the study was retrospective, and the composite reference standard was imperfect, diagnostic performance estimates should be interpreted cautiously. Prospective studies are needed to define patient selection and determine whether PET/CT improves patient-centered outcomes.

Early identification of an infectious focus and timely source control are central components of sepsis management in the ICU. Together with appropriate antimicrobial therapy, these measures aim to limit ongoing inflammatory injury and prevent progression to organ dysfunction and shock. However, in a relevant proportion of critically ill patients, conventional diagnostic approaches, including microbiological testing, ultrasound, and standard cross-sectional imaging, fail to localize a source of infection despite persistent clinical and laboratory evidence of sepsis.

^18^F-Fluorodeoxyglucose positron emission tomography combined with CT (FDG PET/CT) is an established imaging modality for oncological staging (1), the evaluation of fever of unknown origin (2), and the diagnosis of large-vessel vasculitis (3). Its use has recently been extended to selected critically ill patients, and observational studies suggest that examinations can be performed safely in patients receiving mechanical ventilation or vasopressor support (4, 5). Nevertheless, FDG PET/CT is used sparingly in the ICU because of logistical complexity, transport-related risks, limited availability, and concerns about cost and resource utilization. Consequently, its clinical value in septic ICU patients, and the scenarios in which it may be most informative, are not yet established.

We therefore conducted a retrospective observational cohort study of FDG PET/CT examinations performed in septic ICU patients at a tertiary university hospital. Scans were requested either for Question A, search for an unknown infectious focus, or for Question B, confirmation or exclusion of a suspected infectious focus. We aimed to describe the spectrum of infectious foci detected and missed, to document procedural safety, and to assess how often imaging findings were followed by changes in diagnostic or therapeutic management.

## MATERIALS AND METHODS

We performed a single-center retrospective observational cohort study of FDG PET/CT scans obtained in adult patients with sepsis treated in the ICUs of the University Medical Center Hamburg-Eppendorf. A total of 108 scans in 103 patients were included. Two patients underwent PET/CT during separate ICU admissions, and three patients underwent two scans during the same ICU stay. The primary unit of analysis was the FDG PET/CT scan. Analyses were performed pragmatically at the scan level because the clinical question, indication, and subsequent diagnostic or therapeutic consequences were documented for each examination. Repeated examinations in the same patient were not modeled using a clustered approach; because only five patients contributed more than one scan, the influence on variance estimates was considered limited, but this is acknowledged as a limitation. Eligible cases were identified from the logbooks of the Department of Nuclear Medicine. Clinical data were extracted from the electronic health record using a standardized data collection form. Variables included demographic characteristics, vital signs, PET/CT findings, laboratory and microbiological results, additional diagnostic procedures, and clinical outcomes. Data were anonymized after extraction.

**Figure 1.**
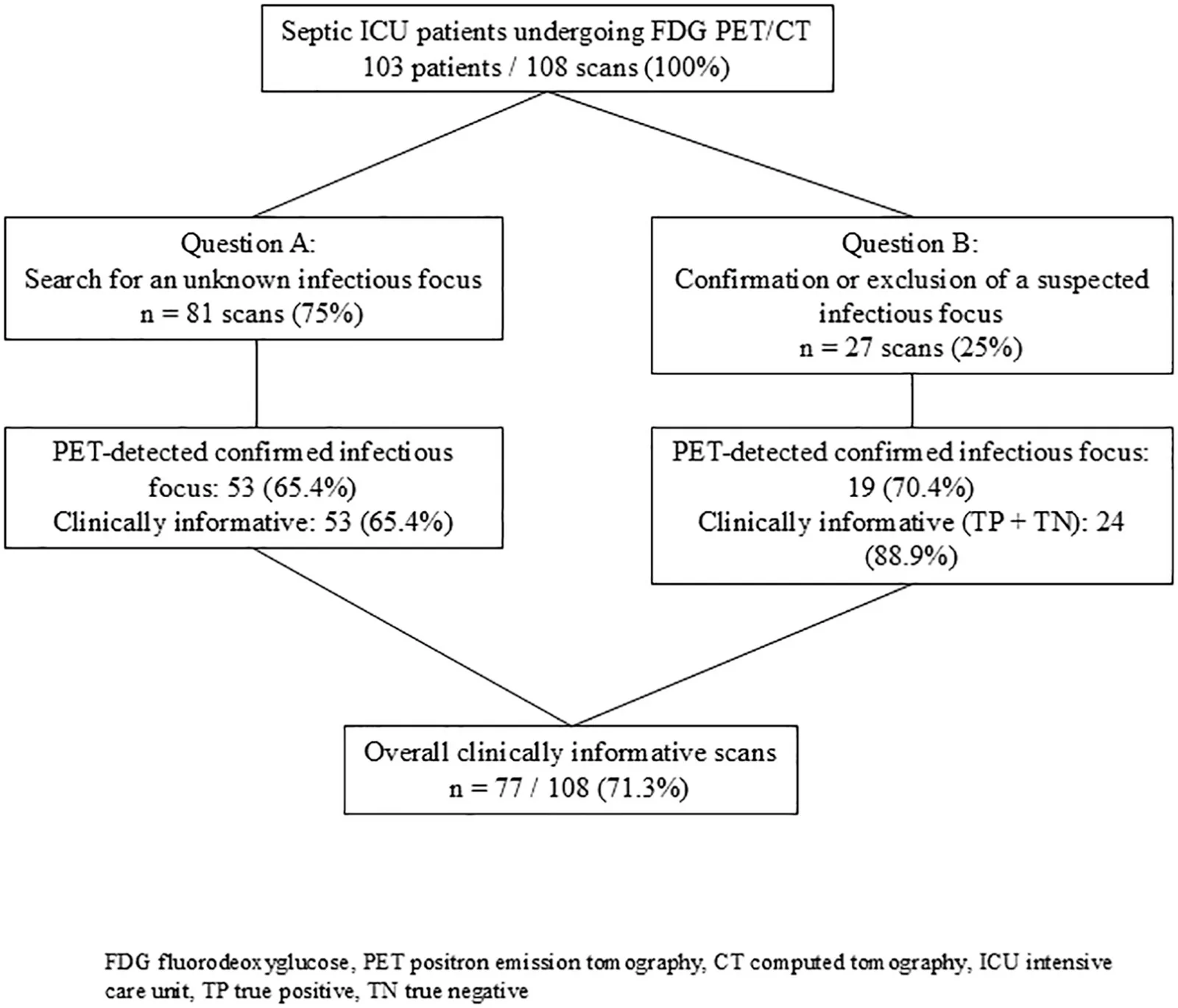
Patient flow and diagnostic classification of ^18^fluorodeoxyglucose positron emission tomography combined with CT (FDG PET/CT) examinations. TP = true positive, TN = true negative.

FDG PET/CT referral was based on multidisciplinary clinical consensus between the attending ICU team and Nuclear Medicine/Radiology, without a predefined study protocol or formal institutional referral algorithm. PET/CT was requested by the treating physician when signs of infection persisted despite inconclusive conventional diagnostics, including laboratory testing, microbiology, radiological imaging, and ultrasound. Examinations were performed for one of two predefined indications: Question A, search for an unknown infectious focus, or Question B, confirmation or exclusion of a suspected infectious focus. Case selection and classification are summarized in **Figure 1**. Diagnostic classification and criteria for clinically informative findings are defined in **Tables 1** and **2**.

**TABLE 1.** Diagnostic Classification of ^18^F-Fluorodeoxyglucose Positron Emission Tomography Combined With CT Scans According to Composite Reference Standard

	Infection Present	No Infection	Total
FDG PET/CT suspicious for infection	72	8	80
FDG PET/CT not suspicious for infection	1	27	28
Total	73	35	108

FDG PET/CT = ^18^F-Fluorodeoxyglucose Positron Emission Tomography Combined With CT.

The reference standard for presence or absence of infection was defined as confirmation by surgery or invasive procedures, microbiological results from sterile sites, concordant findings on follow-up imaging, or clinical course during hospitalization.

**TABLE 2.** Indication for ^18^F-Fluorodeoxyglucose Positron Emission Tomography Combined With CT and Diagnostic Yield

Indication	Scans (*n*)	Positron Emission Tomography-Detected Confirmed Infectious Focus, *n* (%)	Clinically Informative, *n* (%)
Search for an unknown infectious focus (Question A)	81	53 (65.4)	53 (65.4)
Confirmation or exclusion of a suspected infectious focus (Question B)	27	19 (70.4)	24 (88.9)
Total	108	72 (66.7)	77 (71.3)

A scan was considered clinically informative if it identified at least one subsequently confirmed infectious focus in Question A, or if it confirmed or excluded infection at the predefined suspected site in Question B.

Technical aspects of image acquisition and interpretation have been described previously (5, 6). In brief, [18F]FDG PET/CT imaging was performed according to institutional clinical routine. A standard administered activity of approximately 350 MBq was used. All scans were performed after a fasting period of at least 4 hours, with blood glucose levels below 200 mg/dL at the time of tracer injection. Tracer injection was performed at the bedside in the ICU. Image acquisition was conducted on a PET/CT scanner (Gemini GXL10; Philips Medical Systems, Best, the Netherlands). An uptake interval of 60–90 minutes after injection was maintained whenever feasible under ICU conditions. Contrast-enhanced CT was performed as previously described when no contraindications, such as impaired renal function, were present (5, 6).

All patients were accompanied by a critical care physician and a nurse during transport and scanning. The mean time spent outside the ICU was 108 minutes (range, 50–252 min). No transport- or procedure-related adverse events were recorded.

Continuous variables are reported as median and interquartile range. Categorical variables are reported as counts and percentages. Comparisons between scans with and without PET-detected infectious foci were performed using the Mann-Whitney *U* test for continuous variables and Fisher exact test for categorical variables. *p* values are reported descriptively; no adjustment for multiplicity was performed because all group comparisons were exploratory. Diagnostic performance metrics were calculated from the composite reference standard and are reported with two-sided 95% Wald CIs; upper interval limits exceeding 100% were truncated at 100%. For the C-reactive protein (CRP) comparison, the Hodges-Lehmann estimate of the median difference with 95% CI was reported. Analyses were considered exploratory because of the retrospective design, selected cohort, small number of repeated scans, and heterogeneous reference standard.

The study was registered with the Ethics Committee of the Hamburg Chamber of Physicians (WF-081/20, date May 6, 2020, study title: “18FDG-PET/CT in critically ill patients: Are the findings helpful in predicting the patient’s future clinical course, and do they have therapeutic implications? A retrospective analysis.”) and performed in accordance with the Declaration of Helsinki (World Medical Association 2024).

## RESULTS

### Study Population

A total of 108 FDG PET/CT scans were performed in 103 patients with sepsis. Baseline characteristics, treatment at the time of imaging, length of stay, and outcomes are summarized in **Table 3**, and patient flow and diagnostic classification are shown in Figure 1. Most patients were receiving antimicrobial therapy, and a substantial proportion required mechanical ventilation and/or vasopressor support. Median time from ICU admission to imaging was 14 days (interquartile range [IQR], 7–32 d). ICU mortality was 29.1%.

**TABLE 3.** Baseline Characteristics According to ^18^F-Fluorodeoxyglucose Positron Emission Tomography Combined With CT Detection of an Infectious Focus

Characteristics	PET Detected Infectious Focus	No PET-Detected Infectious Focus	*p*
Scans, *n*	72	36	—
Age, yr	60 (50–72)	68 (45–74)	0.372
Male sex, *n* (%)	59/72 (81.9)	17/36 (47.2)	< 0.001
Antibiotic therapy at scan, *n* (%)	68/72 (94.4)	30/36 (83.3)	0.081
Antifungal therapy at scan, *n* (%)	21/72 (29.2)	8/36 (22.2)	0.497
Mechanical ventilation, *n* (%)	39/72 (54.2)	16/36 (44.4)	0.415
Vasopressor therapy, *n* (%)	40/72 (55.6)	20/36 (55.6)	1.000
Mechanical ventilation or vasopressor therapy, *n* (%)	48/72 (66.7)	23/36 (63.9)	0.831
C-reactive protein, mg/L	106.5 (65.8–178.0)	66.0 (29.5–137.8)	0.018
Leukocyte count, /nL	11.8 (8.2–17.0)	10.1 (7.3–15.5)	0.176
Procalcitonin, ng/mL	1.88 (0.59–6.04)	2.12 (0.76–8.52)	0.665
Core temperature, °C	37.6 (37.0–38.2)	37.4 (36.9–38.0)	0.324
Time from ICU admission to ^18^F-Fluorodeoxyglucose PET/CT, d	12 (7–38)	18 (8–38)	0.525
ICU length of stay, d	32 (19–72)	44 (18–98)	0.374
Hospital length of stay, d	55 (37–92)	54 (39–98)	0.625
ICU mortality, *n* (%)	19/72 (26.4)	11/36 (30.6)	0.655

CRP = C-reactive protein, PET = positron emission tomography.

Values are reported per scan. Continuous variables are shown as median (interquartile range); categorical variables are shown as *n*/N (%). PET-detected infectious focus was defined according to whether ^18^F-fluorodeoxyglucose PET/CT detected a subsequently confirmed infectious focus. Comparisons were performed using the Mann-Whitney *U* test for continuous variables and Fisher exact test for categorical variables. *p* values are descriptive and were not adjusted for multiplicity.

### Overall Diagnostic Classification

FDG PET/CT was suspicious for infection in 80 of 108 scans (74.1%) and not suspicious for infection in 28 scans (25.9%). According to the composite reference standard, 72 scans were classified as true positive, eight as false-positive, 27 as true negative, and one as false negative. The resulting diagnostic classification is shown in Table 1.

Based on the composite reference standard, apparent sensitivity was 98.6% (95% CI, 96.0–100.0%), specificity 77.1% (95% CI, 63.2–91.1%), positive predictive value 90.0% (95% CI, 83.4–96.6%), and negative predictive value 96.4% (95% CI, 89.6–100.0)%.

### Indication for FDG PET/CT and Diagnostic Yield

Scans were requested for two predefined clinical indications: Question A, search for an unknown infectious focus, and Question B, confirmation or exclusion of a suspected infectious focus. The distribution of indications, proportion of positive scans, and proportion considered clinically informative are shown in Table 2. For Question A, scans were considered clinically informative when at least one infectious focus was confirmed during the subsequent clinical course. For Question B, both confirmation and reliable exclusion of infection at the predefined suspected site were considered clinically informative.

### Spectrum of Infectious Foci

Across all scans, the most frequent categories of confirmed infectious foci were pulmonary infections, abscesses, and vascular graft or foreign-body–associated infections. Cardiac and musculoskeletal infections were less common. False-positive findings occurred most frequently in gastrointestinal and musculoskeletal categories. A condensed overview of major infection categories is provided in **Table 4**; detailed anatomical breakdowns are shown in **Supplemental Tables 1–3** (https://links.lww.com/CCX/B671). Microbiological findings were available for a subset of confirmed infectious foci and are summarized in **Supplemental Table 7** (https://links.lww.com/CCX/B671). Focus-specific microbiology was documented most often for pulmonary infections and abscesses, whereas many foci were confirmed by radiology, operative findings, or clinical course rather than by culture.

**TABLE 4.** Major Categories of Infectious Foci Detected

Category	True Positive, *n* (%)	False Positive, *n* (%)
Pulmonary	43 (37.7)	4 (12.9)
Abscess	17 (14.9)	4 (12.9)
Vascular graft/foreign body	12 (10.5)	6 (19.4)
Cardiac	4 (3.5)	1 (3.2)
Musculoskeletal	13 (11.4)	6 (19.4)
Gastrointestinal	7 (6.1)	8 (25.8)
Other	18 (15.8)	2 (6.4)

This table summarizes major categories of individual positron emission tomography findings classified as true positive or false positive. Categories are condensed for readability; detailed anatomical distributions and targeted/incidental findings are provided in Supplemental Tables 1–3 (https://links.lww.com/CCX/B671). Percentages refer to the respective column totals. Totals exceed the number of scans because multiple infectious foci could be present in a single patient.

### Association With Inflammatory Markers

CRP concentrations were higher in scans with a PET-detected infectious focus than in scans without such a finding (median, 106.5 mg/L [IQR, 65.8–178.0 mg/L] vs. 66.0 mg/L [IQR, 29.5–137.8 mg/L]; Mann-Whitney *U* test; *p* = 0.018). The Hodges-Lehmann estimate of the median difference was 34 mg/L (95% CI, 8–62 mg/L). Median leukocyte count, procalcitonin, and core temperature showed only small observed between-group differences (Table 3). Median biomarker values and patients with markedly elevated inflammatory markers despite negative FDG PET/CT are further detailed in **Supplemental Tables 4** and **5** (https://links.lww.com/CCX/B671).

### Clinical Impact

FDG PET/CT findings were considered clinically informative in 77 of 108 scans (71.3%) (Table 2). Imaging results were frequently followed by additional diagnostic procedures and therapeutic interventions. Noninvasive investigations were initiated after 50 scans (46.3%), and invasive diagnostic or therapeutic procedures after 52 scans (48.1%). In 49 cases (45.4%), invasive procedures confirmed or directly treated infection. Invasive procedures included operations, bronchoscopies, biopsies, endoscopic procedures, drainages, arthroscopies, and TEE followed by surgery (**Table 5**).

**TABLE 5.** Clinical Consequences of ^18^F-Fluorodeoxyglucose Positron Emission Tomography Combined With CT

Consequences	Scans, *n* (%)
Additional noninvasive diagnostics	50 (46.3)
Any invasive diagnostic or therapeutic procedure triggered	52 (48.1)
Invasive procedure confirmed or directly treated infection	49 (45.4)
Antibiotic regimen modified	44 (40.7)
Antibiotics discontinued	4 (3.7)

Procedure types and antimicrobial modification categories are provided in Supplemental Table 6 (https://links.lww.com/CCX/B671).

Changes in antimicrobial therapy occurred after 44 scans (40.7%), most commonly as switching or prolongation of existing regimens. Clinical consequences following FDG PET/CT are summarized in Table 5 and illustrated in **Supplemental Figure 1** (https://links.lww.com/CCX/B671); additional details on procedure counts and antimicrobial modifications are provided in **Supplemental Table 6** (https://links.lww.com/CCX/B671). These data describe observed downstream actions after imaging and do not establish causal effects of PET/CT on management.

## DISCUSSION

Localization of an infectious focus remains challenging in critically ill patients with sepsis when conventional diagnostics fail to explain. FDG PET/CT has become technically feasible in ICU patients, including those requiring mechanical ventilation or vasopressor support (4, 5), yet clinical experience remains limited and heterogeneous, as reflected by several small series and observational reports (4–12) and summarized in a recent systematic review (13). The present cohort therefore reflects a relatively large real-world experience with FDG PET/CT in a highly selected ICU population.

In our institution, PET/CT was typically considered only after standard microbiological and imaging investigations had not identified a convincing source of infection or when a suspected focus could not be confirmed. Within this context, the examination often contributed to diagnostic clarification, either by identifying a previously unrecognized focus or by reducing suspicion for a presumed site of infection. This supports a selective, problem-oriented use rather than routine deployment of PET/CT in septic ICU patients.

As with any sensitive imaging modality, the potential benefits must be balanced against the risk of overdiagnosis. Diagnostic findings can trigger additional invasive procedures, which may ultimately prove unnecessary and expose patients to avoidable harm (14). Although most invasive procedures prompted by PET/CT findings in our cohort confirmed or treated infection, a minority did not. At the same time, delayed or missed source control remains a major determinant of adverse outcomes in sepsis (15), which explains the clinical interest in highly sensitive imaging approaches. Because management changes were heterogeneous and clinically interdependent—integrating imaging findings, microbiology, bedside assessment, and source-control options—/consequences are presented descriptively rather than by multivariable modeling.

Inflammatory markers were only partly helpful for anticipating scan results. CRP was higher in scans with a PET-detected infectious focus, whereas body temperature, leukocyte count, and procalcitonin showed only small separation between groups, consistent with earlier reports (16, 17). The low yield at very low CRP concentrations is compatible with previous observations that FDG PET/CT is more often informative in patients with greater inflammatory activity (18). CRP may therefore help enrich patient selection, but the observed separation does not justify a stand-alone threshold. Corticosteroid therapy may attenuate FDG uptake and contribute to false-negative results (19), whereas ongoing antimicrobial therapy did not appear to substantially reduce diagnostic yield in our cohort, consistent with prior observations (19).

Regarding specific infectious entities, our findings align with established strengths and limitations of FDG PET/CT. Detection of deep abscesses and occult infections is well documented (20, 21), and vascular graft infections appear particularly amenable to PET/CT evaluation when metabolic findings are interpreted together with CT morphology (22, 23). Cardiac infections remain more challenging, especially native valve endocarditis, where physiologic myocardial uptake and tracer heterogeneity may obscure inflammatory lesions (24–28). Prosthetic valve infections tend to be detected more reliably (29). Similarly, infections of cardiac implantable electronic devices may escape detection in the absence of focal inflammatory activity, despite generally high specificity (30), and echocardiography, particularly transesophageal imaging, remains essential in this setting (31).

Musculoskeletal findings require cautious interpretation because FDG uptake does not reliably distinguish infection from sterile inflammatory processes (32). PET/CT may be useful in suspected spondylodiscitis and can complement MRI (33, 34), but both false-positive and false-negative findings occur. Pulmonary uptake presents additional interpretative challenges in ventilated patients, where fibroproliferative or inflammatory changes may mimic infection (12, 35). Given the limited diagnostic performance of chest radiography for pneumonia (36), PET/CT findings should always be interpreted alongside microbiology, clinical evolution, and other imaging modalities.

These data support the use of FDG PET/CT as an adjunctive diagnostic tool in ICU patients with unresolved infection after conventional evaluation, rather than as a routine investigation in sepsis. The retrospective design, strong case selection, and heterogeneous reference standards limit the interpretation of diagnostic performance metrics. The observed association between PET/CT findings and subsequent diagnostic or therapeutic interventions does not establish improved patient outcomes.

Evidence that FDG PET/CT improves management outside the ICU is beginning to emerge. In the [^18^F]FDG-PET-CT compared with CT for persistent or recurrent neutropenic fever in high-risk patients (PIPPIN) trial, conducted in hematologic patients with persistent or recurrent neutropenic fever, PET/CT increased antimicrobial rationalization compared with conventional CT (82% vs. 65%; *p* = 0.033) (37). The accompanying economic analysis found the approach cost-effective in that population (38). These findings provide a useful reference point, but they cannot be directly extrapolated to septic ICU patients.

Future prospective multicenter studies should enroll ICU patients with persistent sepsis after predefined negative conventional diagnostics, document PET/CT-driven diagnostic and therapeutic actions prospectively, and evaluate patient-centered endpoints including time to source identification, antimicrobial exposure, invasive procedures, ICU length of stay, and mortality. Technological advances, particularly long axial field-of-view PET/CT systems, may facilitate imaging in critically ill patients by reducing acquisition time and logistical burden (39, 40), but whether this will translate into broader clinical adoption or improved outcomes is unknown.

## LIMITATIONS

Several limitations should be acknowledged. The study reflects practice at a single tertiary center with established access to PET/CT imaging, and local referral patterns as well as logistical considerations may therefore have influenced case selection. Because PET/CT was typically used only after inconclusive conventional diagnostics, the cohort represents a population with persistent diagnostic uncertainty after standard workup, rather than the broader ICU sepsis population. The unit of analysis was the scan, and five patients contributed more than one scan. Repeated examinations in the same patient were not modeled using a clustered approach; although the influence on variance estimates is likely limited, this remains a methodological limitation.

In addition, confirmation of infectious foci relied on a heterogeneous and imperfect composite reference standard, including imaging follow-up, microbiology, operative findings, and clinical evolution. Because subsequent diagnostic procedures and clinical interpretation could be influenced by the PET/CT findings themselves, imperfect reference standard bias and incorporation bias cannot be excluded. Diagnostic performance metrics should therefore be interpreted cautiously. Microbiological confirmation was not available for all infectious foci; organism-level data should therefore be interpreted as descriptive. Corticosteroid exposure at the time of imaging could not be reliably reconstructed for all scans and was therefore not included in the comparative analysis. Finally, the study focused on diagnostic performance and clinical decision-making rather than patient-centered outcomes; potential effects on prognosis, resource utilization, or antimicrobial exposure were not addressed and require prospective evaluation.

## CONCLUSIONS

In this retrospective single-center cohort, FDG PET/CT provided clinically useful diagnostic information in selected critically ill patients with unresolved infection after conventional evaluation. The technique was particularly helpful for identifying occult infectious foci or reducing diagnostic uncertainty in complex cases, although false-positive findings and interpretative challenges remain. Given the selective referral pattern and absence of outcome data, these findings should be interpreted cautiously. FDG PET/CT is best regarded as a complementary, problem-oriented diagnostic tool rather than a routine investigation in ICU sepsis. Prospective studies are needed to define optimal patient selection, assess cost-effectiveness, and determine whether its use translates into measurable improvements inpatient-centered outcomes.

## ACKNOWLEDGMENTS

We would like to express our sincere thanks to Prof. Dr. Antonia Zapf of the Institute for Medical Biometry and Epidemiology at the University Medical Center Hamburg-Eppendorf for her assistance and feedback regarding the statistical methodology.

## Supplementary Material
