# Supplementary material for: 18F-Fluorodeoxyglucose Positron Emission Tomography Combined With CT in Unresolved ICU Infections: Diagnostic Yield and Clinical Consequences in a Retrospective Cohort
Source: Crit Care Explor. 2026 Aug 25;8(9):e1467. doi: 10.1097/CCE.0000000000001467 (PMC13506234; doi:10.1097/CCE.0000000000001467)
Supplement: Supplementary file 1 [file cc9-8-e1467-s001.pdf]

FDG PET/CT in Unresolved ICU Infections: Diagnostic Yield and Clinical Consequences in a Retrospective Cohort

Supplemental Digital Content

Table of contents

Supplemental Figure 1. Observed clinical consequences following FDG PET/CT 3

Supplemental Table 1. Detailed anatomical distribution of findings for Question A 4

Supplemental Table 2. Targeted suspected sites for Question B 5

Supplemental Table 3. Incidental findings for Question B 6

Supplemental Table 4. Inflammatory markers according to PET/CT detection of an infectious focus 7

Supplemental Table 5. Patients without PET-detected infectious focus and markedly elevated inflammatory markers 8

Supplemental Table 6. Invasive procedures and antimicrobial therapy modifications 9

Supplemental Table 7. Microbiological findings according to infection category 10

Supplemental Figure 1. Observed clinical consequences following FDG PET/CT

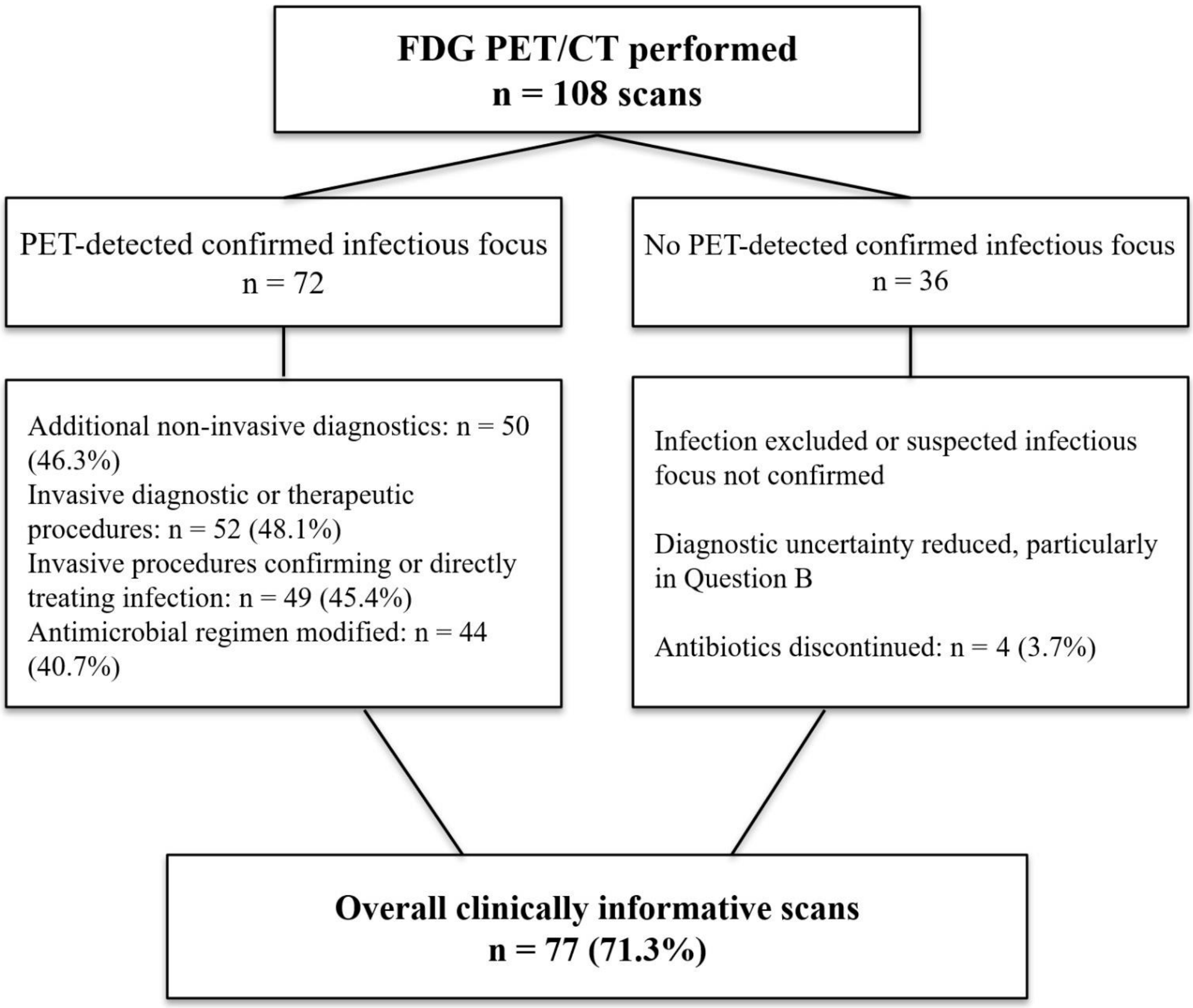

Observed downstream actions after 108 FDG PET/CT scans. Findings were followed by additional non-invasive diagnostics in 50 scans, invasive diagnostic or therapeutic procedures in 52 scans, antimicrobial modification in 44 scans, and antibiotic discontinuation in 4 scans. Overall, 77 scans were considered clinically informative. Numbers are descriptive and do not imply causal attribution.

Supplemental Table 1. Detailed anatomical distribution of findings for Question A (unknown focus)

**Category**	**True positive (n)**	**False positive (n)**	**Total**
Pulmonary (pneumonia/empyema)	33	2	35
Abscess	14	4	18
Joint / arthritis	11	3	14
Gastrointestinal	7	5	12
Foreign body / device	6	2	8
Decubitus	5	1	6
Infected hematoma	3	3	6
Pancreatitis	3	2	5
Lymph nodes	2	1	3
Spondylodiscitis	1	2	3
Endocarditis (prosthetic)	2	0	2
Vascular infection	1	1	2
Other	4	8	12

Supplemental Table 2. Targeted suspected infection sites and diagnostic classification in patients scanned for suspected focus (Question B)

**Infection category**	**TP**	**FP**	**TN**	**FN**	**Sites evaluated**
Foreign body infection, total	6	0	8	3	17
- Vascular prosthesis	6	0	6	0	12
- Pacemaker / TIPS	0	0	2	3	5
Spondylodiscitis	1	0	3	1	5
Endocarditis	0	0	1	1	2
Abscess	0	0	2	0	2
Aortitis	0	0	1	0	1
Other	0	1	1	0	2

TP, true positive; FP, false positive; TN, true negative; FN, false negative; TIPS, transjugular intrahepatic portosystemic shunt; TEE, transesophageal echocardiography.

Supplemental Table 3. Incidental infectious findings in patients scanned for suspected focus (Question B)

**Category**	**True positive**	**False positive**	**Total**
Pulmonary	10	0	10
Infected hematoma	3	1	4
Abscess	3	0	3
Gastrointestinal	0	3	3
Foreign body / device	0	2	2
Joint	0	2	2
Vascular infection	1	1	2
Pancreatitis	1	1	2
Other	1	2	3

Supplemental Table 4. Inflammatory markers according to FDG PET/CT result

**Marker**	**PET-detected infectious focus (median)**	**No PET-detected infectious focus (median)**
Leukocyte count (/nL)	11.8	10.1
CRP (mg/L)	106.5	66.0
PCT (ng/mL)	1.9	2.1
Temperature (°C)	37.6	37.4

PCT, Procalcitonin; CRP, C-reactive protein.

Supplemental Table 5. Patients without PET-detected infectious focus and markedly elevated inflammatory markers

**Criterion**	**Number of patients**
PCT ≥ 4 ng/mL	9
CRP ≥ 100 mg/L	15
Cardiac source finally diagnosed	4
ICU mortality	6

PCT, Procalcitonin; CRP, C-reactive protein; ICU, Intensive Care Unit

Supplemental Table 6. Invasive procedures and antimicrobial therapy modifications triggered by FDG PET/CT findings

**Category**	**Number**
Operations	22
Bronchoscopy	22
Biopsy (microbiology)	12
Endoscopy	5
Arthroscopy	2
Drainage	3
TEE + surgery	2
Antibiotics initiated	2
Antibiotics switched	16
Antibiotics prolonged	15
Antibiotics stopped	4
Other/uncategorized antibiotic modification:	7

Procedure counts are reported per infectious finding, whereas antimicrobial therapy modifications are reported per scan. More than one procedure could be triggered by a single FDG PET/CT examination. TEE, transesophageal echocardiography.

Supplemental Table 7. Confirmed infectious foci and microbiological findings

**Infection category**	**Confirmed foci, n**	**Focus-specific microbiology documented, n**	**Main organisms detected**
Pulmonary infection / pneumonia	40	21	*Pseudomonas aeruginosa* (5); *Candida* spp. (4); *Staphylococcus aureus* (3); coagulase-negative staphylococci (2); *Enterobacter* spp. (2); *Enterococcus* spp. (2)
Other	15	4	*Staphylococcus aureus* (1); *Candida* spp. (1); *Pseudomonas aeruginosa* (1); coagulase-negative staphylococci (1)
Foreign body / device-associated infection	12	2	Coagulase-negative staphylococci (1); *Citrobacter* spp. (1)
Abscess	11	6	*Enterococcus* spp. (2); *Staphylococcus aureus* (2); *Clostridium* spp. (1); coagulase-negative staphylococci (1); *Nocardia farcinica* (1)
Joint infection / arthritis / effusion	10	3	*Candida* spp. (1); *Staphylococcus aureus* (1); organism not specified (1)
Gastrointestinal infection / colitis	6	0	No focus-specific microbiology documented
Pressure ulcer	5	0	No focus-specific microbiology documented
Infected hematoma	5	1	*Staphylococcus aureus* (1)
Pleural/pulmonary infection other than pneumonia	4	0	No focus-specific microbiology documented

Infection category	Confirmed foci, n	Focus-specific microbiology documented, n	Main organisms detected
Endocarditis	2	0	No focus-specific microbiology documented
Vascular infection	2	0	No focus-specific microbiology documented
Lymph node involvement	2	0	No focus-specific microbiology documented
Pancreatitis	2	0	No focus-specific microbiology documented
Spondylodiscitis	2	1	*Staphylococcus aureus* (1)

Confirmed foci refer to individual PET findings classified as true-positive in the focus-level dataset. Focus-specific microbiology was counted when culture-based confirmation or a specific pathogen was documented in the diagnostic workup of that focus. Microbiological confirmation was not available for all foci because the composite reference standard also included operative findings, radiology, follow-up imaging, and clinical course. Organism counts refer to documented focus-level microbiology and may not capture all scan-level cultures obtained during the ICU stay. In some cases, microbiological confirmation was documented without organism-level information in the focus-level dataset.

Listed organisms are descriptive and may not sum to the number of microbiologically documented foci because cultures could be polymicrobial, and only organisms explicitly documented at focus level were extracted.
